# Rapid screening for phenotype-genotype associations by linear transformations of genomic evaluations

**DOI:** 10.1186/1471-2105-15-246

**Published:** 2014-07-19

**Authors:** Jose L Gualdrón Duarte, Rodolfo JC Cantet, Ronald O Bates, Catherine W Ernst, Nancy E Raney, Juan P Steibel

**Affiliations:** Departamento de Producción Animal, Facultad de Agronomía, UBA-CONICET, Buenos Aires, Argentina; Department of Animal Science, Michigan State University, East Lansing, MI USA; Department of Fisheries and Wildlife, Michigan State University, East Lansing, MI USA

**Keywords:** Genome wide association, Marker variance, Pig genotype

## Abstract

**Background:**

Currently, association studies are analysed using statistical mixed models, with marker effects estimated by a linear transformation of genomic breeding values. The variances of marker effects are needed when performing the tests of association. However, approaches used to estimate the parameters rely on a prior variance or on a constant estimate of the additive variance. Alternatively, we propose a standardized test of association using the variance of each marker effect, which generally differ among each other. Random breeding values from a mixed model including fixed effects and a genomic covariance matrix are linearly transformed to estimate the marker effects.

**Results:**

The standardized test was neither conservative nor liberal with respect to type I error rate (false-positives), compared to a similar test using Predictor Error Variance, a method that was too conservative. Furthermore, genomic predictions are solved efficiently by the procedure, and the *p*-values are virtually identical to those calculated from tests for one marker effect at a time. Moreover, the standardized test reduces computing time and memory requirements.

The following steps are used to locate genome segments displaying strong association. The marker with the highest − log(*p*-value) in each chromosome is selected, and the segment is expanded one Mb upstream and one Mb downstream of the marker. A genomic matrix is calculated using the information from those markers only, which is used as the variance-covariance of the segment effects in a model that also includes fixed effects and random genomic breeding values. The likelihood ratio is then calculated to test for the effect in every chromosome against a reduced model with fixed effects and genomic breeding values. In a case study with pigs, a significant segment from chromosome 6 explained 11% of total genetic variance.

**Conclusions:**

The standardized test of marker effects using their own variance helps in detecting specific genomic regions involved in the additive variance, and in reducing false positives. Moreover, genome scanning of candidate segments can be used in meta-analyses of genome-wide association studies, as it enables the detection of specific genome regions that affect an economically relevant trait when using multiple populations.

**Electronic supplementary material:**

The online version of this article (doi:10.1186/1471-2105-15-246) contains supplementary material, which is available to authorized users.

## Background

The availability of high density genotypes of single nucleotide polymorphism (SNP) markers for plants and livestock species, in conjunction with phenotypic data for complex traits, allows the calculation of: 1) estimates of genomic breeding values (GEBVs) [1, 2] for genomic evaluation [3], and 2) estimates of the effects of genomic regions associated with the genetic variability in genome wide association studies (GWAS) [2, 4, 5].

There is an increasing number of GWAS data sets analyzed by mixed models and multiple testing procedures [6], after fitting all individual effects of genomic regions into the model [4]. The model may be difficult to fit when both, the number of individuals and SNP effects, are large. We propose to use a linear transformation of genomic breeding values to estimate the marker effects from a simpler equivalent mixed model, and then testing those effects using a standardized test statistic that employs the variance (rather than prediction error variance) of the same effects.

The method of *genomic selection* proposed by Meuwissen et al. [7] to estimate GEBVs starts by fitting the SNP effects to a given data set. Next is to estimate GEBV of any individual using its genotype (SNP), by adding across the entire genome those solutions corresponding to the individual's SNP. The mixed model employed conveys vectors of fixed effects, and random effects of markers or SNPs ( ***g*** ) assumed to be normally distributed with null mean and a covariance matrix proportional to the identity matrix times the variance of SNP effects . Errors are assumed to be Gaussian, independent and identically distributed with null mean and covariance matrix . An equivalent mixed model discussed by Garrick [8] and Stranden [9] is fitted after the linear transformation ***a*** = ***Z g*** where ***a*** is a random vector of breeding values, and ***Z*** an incidence matrix that relates elements in ***a*** to those in ***g***. Each column of ***Z*** is associated with a given SNP and the elements are standardized by functions of SNP allele frequencies and by the total number of SNP. It is worth noting that the same ***Z*** is used in our implementation of the model of Meuwissen et al. [7] to relate the vector of marker effects in ***g*** to the data phenotypes. Moreover, GEBVs in the equivalent model have variance-covariance matrix . The procedure requires that the variances are equal, i.e. . Once the equivalent model is fit, SNP effects are calculated by the transformation ***g*** = ***Z***^'^***G***^− **1**^***a***, and individual SNP effects in ***g*** are divided by the square root of its variance (Var( ***g***_*j*_ )) to get the so called SNP_*ej*_ test statistics. We also provide a formula to calculate Var( ***g***_*j*_ ) without having to fit the model with SNP effects. The next step is to select genome segments that may be highly associated with the genetic variability of the trait for each chromosome. In doing so, we look for the SNP having the highest value of minus the logarithm of the *p*-value throughout the chromosome. Once the SNP is located, a segment of one Mb to the left and one to the right is defined, and a relationship matrix is calculated using only the information from those markers. The relationship matrix is used as the proportional variance-covariance of the segment effects in a model that also includes fixed effects and random GEBVs. In a final step, the likelihood ratio is calculated to test the significance of the largest effect segment of each chromosome by comparing against a reduced model with fixed effects and GEBVs. The critical value (size of the test) is adjusted by the Bonferroni correction. The algorithm not only delivers genome wide associations and genomic predictions efficiently, but it also minimizes computing time and memory requirements. Moreover, the specific variance of the SNP effects is used in calculating the test, thus taking into account the amount of information of any given marker. Instead, other testing approaches rely on a prior variance or a constant estimate of the additive variance.

## Methods

### Dataset

The experimental population was raised at the Michigan State University Swine Teaching and Research Farm, East Lansing, MI [10]. Parents from the initial generation (F_0_) were four Duroc boars mated to 15 Pietrain sows by artificial insemination. From all resulting F_1_ animals, 50 females and 6 males (progeny of 3 F_0_ sires) were selected as parents for the F_2_ generation, by avoiding full or half sib matings. A total of 1,259 F_2_ piglets were born alive from 142 litters out of 11 farrowing groups. Phenotypic data for growth, carcass merit and meat quality traits were collected for approximately 950 F_2_ pigs (for more details refer to Edwards et al. [10, 11]). Data used for the study were measures of the growth trait *13 week tenth rib backfat (mm)* (bf10_13wk). The trait was chosen as it displays a sizable heritability (0.42) and a normal distribution. Animal protocols were approved by the Michigan State University All University Committee on Animal Use and Care (AUF# 09/03-114-00).

### Genotyping and data editing

DNA was isolated from white blood cells using standard procedures as previously described for this population [10]. Quantity and quality of DNA samples were determined using a Qubit fluorometer (Invitrogen by Life Technologies, Carlsbad, CA, USA). The experimental population was genotyped with two marker SNP panels. 1) 411 animals were genotyped (4 F_0_ Duroc boars, 15 F_0_ Pietrain sows, 6 F_1_ males, 50 F_1_ females and 336 F_2_ pigs) with a commercial panel, the Illumina PorcineSNP60 beadchip (60 K) [12] and 2) 612 F_2_ animals were genotyped with a second panel composed of a 9 K tagSNP set referred to as the GeneSeek Genomic Profiler for Porcine LD (GGP-Porcine, GeneSeek a Neogen Company, Lincoln, NE) [13] . A set of 5,350 SNP out of *M* = 62,163, were eliminated from all analyses as their physical positions were unknown. Mendelian inconsistencies (≤0.01%) were taken as missing genotypes, and 21 animals (1 F_1_ and 20 F_2_) with more than 10% of SNP missing were not used for any analysis. By similar considerations, 2,978 SNP were removed from the analyses as they had more than 10% missing data. Additionally, 9,877 SNP were excluded as their minor allele frequency (MAF) was below 0.01. This editing procedure followed that of Badke et al. [14] and Gualdrón et al. [15], and the program PLINKv1.07 [16] was used for the task. F_2_ animals genotyped with the 9 K panel were imputed to 60 K following procedures discussed by Gualdrón et. al [15], by means of the software AlphaImpute [17], resulting in imputation accuracy of around 0.99 [15]. Genotypes imputed in the F_2_ had a second editing procedure by MAF < 0.01, which excluded 759 virtually monomorphic SNP. The editing policies and genotype imputation resulted in a data set with records from 1002 pigs (F_0_, F_1_ and F_2_) having 44,055 SNP per animal.

### Estimation of genomic relationship matrix

The genomic relationship matrix was estimated from observed and imputed high density (~44 K) SNP genotypes. Genotypes were expressed as allelic dosage [13, 15], such that genotypes were entered into a marker matrix ***M*** of dimension (*n* × *m*), where *n* is the number of animals and *m* the number of SNP, having elements in the interval [0, 2], i.e. the count of the allele used as reference. In the sequel, we will use the sub index *i* to refer to the individual. Matrix ***M*** was standardized to matrix ***Z*** that has generic elements equal to


Elements of ***Z*** are then calculated by subtracting twice the frequency of the reference allele at the *j*th marker (*p*_*j*_), to the corresponding element of ***M*** [18], and then dividing the resulting difference by the square root of the expected variance 2*p*_*j*_(1 − *p*_*j*_) of each element in the column multiplied by the number of columns (*m*) in ***M***. The allele frequency *p*_*j*_ was calculated from the F_0_ generation (19 animals). The genomic relationship matrix was finally calculated as:
1

### Prediction model

Using the genomic relationship matrix from equation (1), the centered animal model for genomic evaluation can be written as:
2a

where ***y*** is the phenotypic vector containing the data on *13-week tenth rib backfat* (*mm*), ***X*** is the incidence matrix that relates records to the fixed effects of sex in **β**, vector ***a*** contains the random breeding values such that , ***e*** is the random error vector such that , and ***I*** is the identity matrix. Variance components were estimated with REML using the regress version 1.3-10 R package [19].

Following Stranden et al. [9] an equivalent model to (2a) is
2b

Every element in (2b) is defined as before except for the vector ***g*** of SNP effects. To show that (2a) and (2b) are equivalent models, we employ the fact that ***a*** = ***Z g***. Then, the variances of ***a*** and ***g*** are related in the following manner:


Necessary conditions for models (2a) and (2b) to be equivalent (Henderson, 1984) are that ***G*** = ***Z Z*** ' and .

### Variance of SNP effects

In this section, we describe the algorithm to calculate the variance of the estimated SNP effects ***g***. The SNP effects were obtained from a linear transformation of breeding values in  [4, 9, 20, 21], as follows:
3

The last step results from the fact that model equivalence involves . Now, from equation (3)  is obtained as follows:
4

Now, we know that the predictor error variance (PEV) of  from model (2a) is equal to:


So that


Matrix ***C***^***aa***^ results from inverting the coefficient matrix of the mixed model equations [22] such that:


Then, on replacing with the latter expression into  (displayed in (4)), we have:
5

Expression (5) results in a large matrix of dimension (*m* × *m*) with *m* the number of SNP. However, we only need its diagonal elements. Also notice that the first term in (5), ***Z*** ' ***G***^− **1**^ ***Z***, can be computed and stored to be reused for the different traits, whereas ***C***^***aa***^ has to be computed for each trait.

### Standardization of SNP effects (SNP_*ej*_)

The estimated SNP effects in (3) were standardized by dividing with their corresponding  obtained from (5) as follows:
6

### P-values and genome screening

The *p*-values were assessed as 1 minus the cumulative probability density of the absolute value of *SNP*_*e j*_, a number that was then multiplied by 2 so as to obtain:


where *Φ*(*x*) is the cumulative density function of the normal distribution for the random variable *x*. When analyzing the trait *13 week tenth rib backfat (mm)*, the *p*-values for each SNP were plotted across the genome as –Log_10_ (*p*-value) using the physical position of the SNP in Mega-bases (Mb).

### Standardization of SNP effects using the PEV of the marker

A second standardization of the *j*^th^ SNP effect (3) was performed using the PEV  as follows:
7

As discussed above, . The *p*-values and genome screening for *SNP*_*ep j*_ were assessed and plotted in the same fashion as for *SNP*_*e j*_.

### Simulation

A plasmode simulation was performed to compare how the standardized values *SNP*_*e j*_ and *SNP*_*ep j*_ affected the nominal size of the test for the effect to be equal to zero. Data on 928 animals with 44,055 SNP each were used for the study, and the 1018 SNP on chromosome 18 were reshuffled. Two scenarios were considered: 1) Dependency: rows of the genotype matrix were permuted for columns corresponding to SNP on chromosome 18, thus keeping Linkage Disequilibrium (LD) within chromosomes but breaking the relationship between genotypes and phenotypes for the 1018 SNP on the chromosome. 2) Independency: the genotype of any animal was permuted independently by marker (resulting in linkage equilibrium, or LE between markers) for those SNP on chromosome 18, and the relationship with the phenotype was broken too. For both scenarios model (2a) was fitted to the data, and two tests were calculated for each scenario: test1 = *SNP*_*ej*_ and test2 = *SNP*_*epj*_. Permutations were repeated 200 times per scenario, and in each permutation the ***G*** matrix was calculated while fitting model (2a). As a result, the heritability of the trait was similar to the original heritability due to relationships in the other 17 chromosomes being kept intact, and *p*-values for those SNP (that are now non-associated) on chromosome 18 were obtained for the different tests. Under the null hypothesis and assuming independence (i.e., SNP are unlinked to the polymorphism controlling the trait), an approach that controls for type I error appropriately [23], the 1018 test *p*-values follow a uniform distribution. Consequently, to estimate the empirical quantiles of the distribution for the null hypothesis, we used a uniform density U ∼ (0, 1) to generate 200 replicated sets for the 1018 *p*-values.

### SNP effects and tests obtained by a single marker model

The SNP effects were tested on a one by one basis. The model approach used for testing purposes is better known as “efficient mixed-model association” (EMMA) [24]. The model included fixed effects of sex and one-marker-at-a-time; random variable was the animal effect with variance-covariance equal to the genomic relationship matrix using all markers, which was calculated as described before. The R package rrBLUP [25] was used for fitting the different models and for calculating the tests and *p*-values.

### Proportion of variance explained by segments with large effect

After the genome screen using model 2a, the SNP with the smallest *p*-values were selected to form SNP segments. These segments were defined by taking all SNP within one Mb upstream and one Mb downstream of the SNP with smallest *p*-value on each chromosome. The size of the segment was chosen using a criterion similar to the one employed by Hayes et al. [4]. The point of change in the rate of decay in linkage disequilibrium in this population was about *r*^2^ = 0.2 at 1 Mb (data not shown), which essentially would imply a minimal contribution to the additive variance from markers located beyond such distance. Moreover, segment sizes about two Mb have been reported to be significant in association studies [20, 26–28]. The proportion of variance associated with each segment was estimated by building a genomic relationship matrix ***G***_1_ (as described in (1)) using all SNPs that belonged to the segment, whereas genomic relationship matrix ***G***_2_ was built using all remaining SNPs. The model fitted can be represented as:
8

where ***a***_1_ is the vector of additive random effects associated with those SNP located in the segment, such that , and ***a***_2_ is the vector of additive random effects associated with all SNPs except those involved with ***a***_1_, such that . Model (8) assesses the proportion of variance explained by the segment of interest (local variance) from the genome variance explained by all SNPs (global variance). The variances estimated in (8) were compared with those estimates from model (2a). Hayes et al. [4] used a similar model to assess the segment variance. Applying either model (8), or the approach of Hayes et al. [4] gave similar estimated variance components. In practice, the advantage of fitting model (8) is that ***G***_2_ is computed by subtracting from ***G*** the columns of ***Z*** related to the segment being tested. Let ***Z***_*s*_ be a matrix having as columns those related to the segment being tested, then . On the contrary, in the model of Hayes et al. [4] *G*is different from segment to segment. Additionally, the calculation of ***G***_1_ and  is fast and involves only those SNPs located in the segment.

To adjust the level of significance for multiple comparisons, a Bonferroni Correction (BC) was performed. In this context, if the pig genome is ~2800 Mb long and the average size of the segment is 2 Mb, there are 1400 segments along the genome with corresponding multiple tests. Thus, for α = 0.05, the BC was equal to 0.05/1400 = 3.571429*e*^−05^ (adjusted α or critical value). Hence, in order to evaluate the significance of the segments, a second *p*-value for the Likelihood Ratio Test (*p* − value_LRT_) was calculated to compare against BC. This *p* − value_LRT_ was assessed as 1 minus the distribution function of a chi-square (χ^2^) random variable with 0.5 degrees of freedom [29, 30] as follows:


where *Ω*(*x*) is the distribution function of a random variable having the χ^2^ as density, and LRT is the Likelihood Ratio Test obtained by contrasting appropriate models.

## Results

### Genome screening

**Figure 1:**
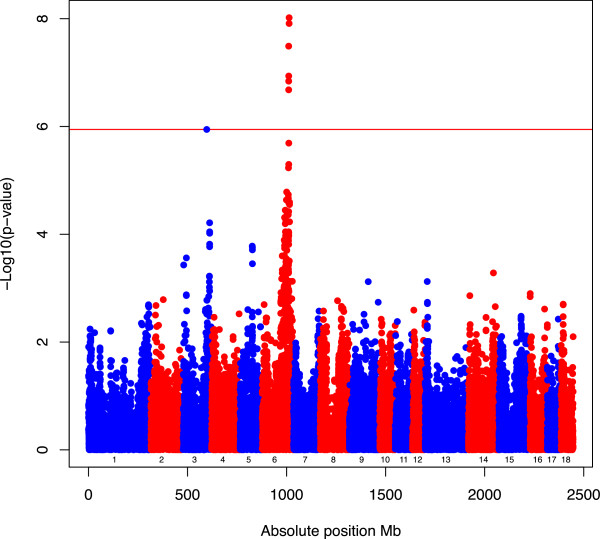
**Manhattan Plot for trait 13-week tenth rib backfat (mm) by standardization SNP**_**ej**_ **.** Genome screening for 44055 SNP using standardization . −log_10_ ( *p*-value ) ( *y* axis ) versus the absolute SNP position in Mb ( *x* axis ). The red line represents a genome-wide significance threshold (*p* < 1.1349 × 10^−6^). Numbers from 1 to 18 represent the chromosome ID.

**Figure 2:**
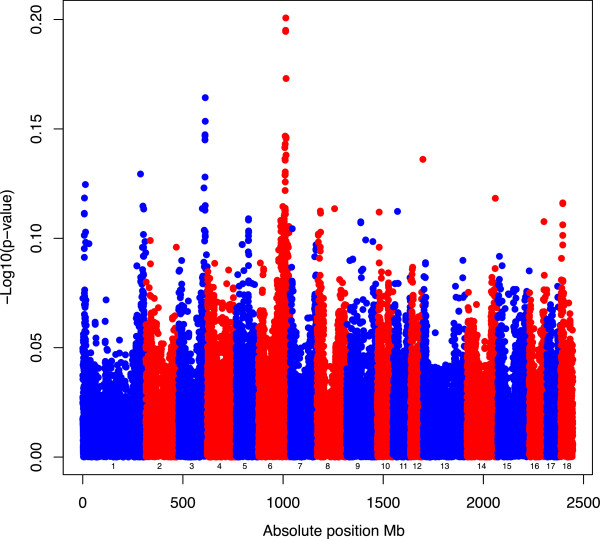
**Manhattan Plot for trait 13-week tenth rib backfat (mm) by standardization SNP**_**epj**_ **.** Genome screening for 44055 SNP using standardization . −log_10_ ( *p*-value ) ( *y* axis ) versus the absolute SNP position in Mb ( *x* axis ). Numbers from 1 to 18 represent the chromosome ID.

The *p*-values of the 44055 SNP were obtained as described in the Methods section. First, the *p*-values for *SNP*_*ej*_, i.e. using , were plotted along the genome (Manhattan plot in Figure 1) to identify genomic positions that are associated with variation in *13-week tenth rib backfat* (*mm*). Large peaks (**−**Log_10_(*p*-value) above 5 can be seen at chromosomes 6 and 3, suggesting noticeable genetic variation for the trait. On the other hand, *p*-values for *SNP*_*epj*_ (i.e. standardized with prediction error variance) were very large, with a maximum − Log_10_( *p*-value2) of 0.20. In essence, the pattern observed in Figure 2 is the result of dividing the non-standardized SNP effects by a constant. Specifically, the normalizing value was [Var (***g***_*j*_) − Var ], with Var (***g***_*j*_) = 2.6768. The use of the square root of the difference between those two values resulted in a practically constant denominator for the test-statistic that was equal to 2.66. Also, a look at Figure 2 suggests signals at chromosomes 1, 12, 14, and 18, a fact that is not observed in Figure 1. However, this might be an artefact of the constant denominator that tends to overestimate the true variability for some SNP, thus resulting in corresponding false positives across the genome.

**Figure 3:**
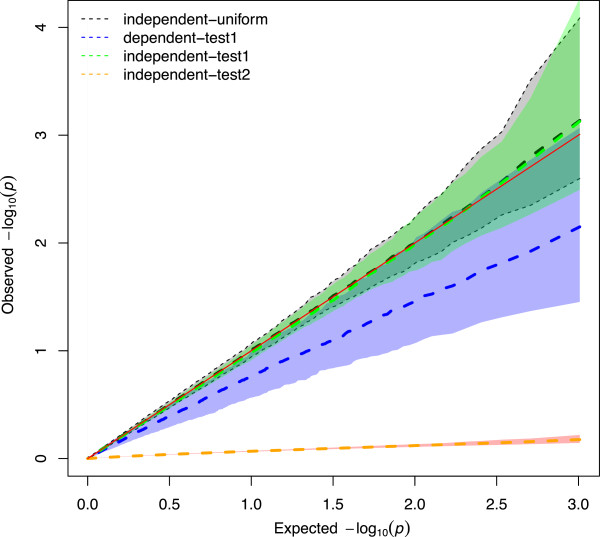
**Quantil-quantil plot of the observed and expected –log(p-values) obtained by simulation.** Reference distribution was an independent and uniform distribution U ∼ (0, 1) for 1018 p-values simulated (black dotted line). Test1(scenario1) = under dependent (LD) and standardization by  (blue dotted line). Test1(scenario2) = under independent (LE) and standardization by  (green dotted line). Test2(scenario2) = under independent (LE) and standardization by PEV (orange dotted line). Each scenario has 1018 *p*-values permuted 200 times. Bands represent confidence intervals of 95% (blue band = test1(scenario1), green band = test1(scenario2), pink band = test2(scenario2).

In order to study the type I error rate of the two proposed tests we performed a plasmode simulation [31]. A plasmode is a dataset created from real data where some of the truth is known. In brief, our plasmode is a simulation that uses reshuffling in a portion of the data as explained in the methods section. We performed a simulation assuming independent SNP, and another one keeping the dependency between SNP (LD structure) intact. Simulation results were plotted into a Quantil-quantil plot graph (Figure 3) using the number –Log(*p*-value) for each case of standardization. First, the *p*-values for test1 (*SNP*_*ej*_) obtained in the scenario under independent SNPs (scenario 2, LE) displayed an identical distribution of *p*-values when obtained by the reference distribution U ∼ (0, 1). In contrast, under dependency (scenario 1, LD) less extreme *p*-values were observed, a fact that was not reflected in a uniform distribution. This is a well known fact in human genetic epidemiology [32], where the implementation of the Bonferroni correction of *p*-values from associated SNP under the assumption of independence results in tests that are too conservative. On the other hand, for test 2 (*SNP*_*epj*_) even *p*-values obtained for independent SNP (scenario 2, LE) displayed a distribution that was too conservative. Furthermore, the results from the dependent scenario (LD) were even more conservative than those from the independent scenario (results not displayed in the Q-Q plot), thus indicating that the use of the square root of  as the denominator of the test-statistic results in a more powerful and not too liberal choice when compared to the use of the square root of PEV = .

### SNP effects and tests obtained by the marker model

The analyses of one SNP tested at a time using the EMMA procedure [24] resulted in *p*-values that were almost identical (Additional file 1) to those of *SNP*_*ej*_ (Additional file 2). The time taken to compute 44055 SNP tests one at a time was 84 minutes. In comparison, the algorithm used to fit model (2a) and to perform the tests of standardized effects took a total time of 29 minutes (CPU and memory: Quad-core 2.7GHz AMD Opteron 8384, 256 GB). This time includes the computation of the ***G*** matrix, the fit of the animal model, the back transformation to calculate the SNP effects, and the calculation of the standard errors that are needed to compute the test-statistics.

### Tests of segment effects

We also compared the results from our proposed method to those obtained with a segment-based likelihood ratio test that has been used by animal breeders [4]. Due to computational demand, we only performed the LRT to test for segment effects. Thus, the SNP with the smallest *p*-values (or highest **−** Log_10_(*p*-values)) on each chromosome were chosen, whereas no segments were tested using LRT for regions with *SNP*_*epj*_ resulting in exceedingly low *p*-values. The three segments from chromosomes with the smallest *p*-values are displayed in Table 1, and the remaining segments from the 15 other chromosomes are shown in the additional files (Additional file 3). All segments measured 2 Mb (1 Mb on each side of the SNP with the smallest *p*-value). The estimates of the variance components and the LogLikelihood obtained from model equation (8) were compared with those from model equation (2a). These results are displayed in Table 2.

**Table 1:** **SNP selected by smallest p-value per chromosome**

SNP-name	Chromosome	Position Mb	-log _10_(p-value)	
ALGA0104402	6	136.08	8.02	0.77
H3GA0010564	3	119.34	5.95	0.48
ALGA0032063	5	61.37	3.78	0.42
ALGA0081287	14	125.98	3.28	0.33
DRGA0011971	13	10.47	3.12	0.36
MARC0022304	9	94.99	3.12	0.42
ALGA0106422	16	111.82	2.90	0.28
ASGA0010464	2	62.15	2.79	0.30
ALGA0111088	8	88.01	2.77	0.48
ASGA0078865	18	10.72	2.70	0.49
ALGA0010607	1	302.88	2.69	0.43
MARC0082230	12	6.14	2.59	0.31
ALGA0045724	7	129.47	2.57	0.41
ASGA0092331	4	138.29	2.52	0.27
ASGA0070227	15	111.82	2.48	0.29
ASGA0077393	17	55.27	2.43	0.32
ASGA0045992	10	7.00	2.42	0.30
ALGA0060793	11	10.50	2.38	0.34

SNP name = SNP marker name, Position Mb = Marker physical position in Mega-Bases, −log_10_(*p*-value) = −Logarithm in base 10 of the smallest *p*-value,  = absolute value of the SNP effect estimated for the trait *13 week tenth rib backfat (mm)*.

**Table 2:** **Variance components and LogLikelihood for models with or without the segment**

Seg-chromosome	6	3	5
**SNP** − **log**_**10**_ **(p-value)**	8.02	5.94	3.78
**Lk_m1**	−1227.938	−1227.938	−1227.938
**Lk_m2**	−1210.800	−1223.178	−1224.540
**LRT**	34.28	9.52	6.80
**p-value**_**LRT**_	1.1 × 10^−9^	6.5 × 10^−4^	3.1 × 10^−3^
**VarE_m1**	3.70	3.70	3.70
**VarA_m1**	2.68	2.68	2.68
**VarE_m2**	3.73	3.67	3.69
**VarA_m2**	1.95	2.42	2.55
**segmVA**	0.70	0.63	0.15
**%segmVA**	0.11	0.09	0.02

**Seg-chromosome** = Number of chromosome where segment is located, **m1** = model(2a) without the segment: *y* = *X*β + *a* + *e*, **m2** = model (8) with the segment ***y*** = ***X*** **β** + ***a***_1_ + ***a***_2_ + ***e***, **SNP** − **log**_**10**_ **(p-value)** = −Logarithm in base 10 of the SNP *p*-value selected to create a segment, **Lk_m1** = −LogLikelihood for m1, **Lk_m2** = −LogLikelihood for m2, **LRT** = Likelihood Ratio Test for m1 and m2, **p-value**_**LRT**_ = *p*-value for LRT, **VarE_m1** = Error variance  of m1, **VarA_m1** = Additive variance  of m1, **VarE_m2** = Error variance  of m2, **VarA_m2** = Additive variance  of m2, **segmVA** = Additive variance segment  of m2, %**segmVa** = Proportion in% of the total variance explained by the segment.

Results from the LRT indicated that the segment on chromosome 6 was significant: *p* − value_LRT ‒ 6_ = 1.133459*e*^−09^, a number smaller than the critical 0.05 Bonferroni threshold for 1400 segments (*P*_critical_ = 0.05/1400 = 3.571429*e*^−05^). On the contrary, the segments located on all other chromosomes were not significant. The proportion of variance explained by the segment from chromosome 6 (−Log(*p*-value) = 8.02) was 11% of the total variance, a fact that was reflected in a similar reduction of the estimated additive variance  in model (8): 1.952 + 0.698 = 2.650. This latter value is close to 2.678, i.e. the estimated value of  from model (2a) (see Table 2). For all other chromosomal segments, the estimated value of  did not decrease to a significant amount.

## Discussion

The main goal of this research was to develop a novel procedure to perform a rapid genome scan, or GWAS analysis, from a genomic evaluation. Moreover, the *sufficient statistics* of our methodology are: the Best Linear Unbiased Prediction (BLUP) of the breeding values from an animal model, ***G*** as the covariance matrix (or ***H*** for a single step evaluation [33]), ***Z*** as the standardized marker effects matrix, variance components, and ***C***^***aa***^. This setting makes the implementation extremely feasible after the genomic evaluation has been performed as discussed by Legarra et al. [33].

### Variance of the SNP effect

First, the SNP effects  were calculated by a linear transformation of  using expression (3). Then, we calculated  using an expression derived from mixed model theory (see (4–5)). Next, we divided  by the square root of  to standardize the effect, and referred the statistics as *SNP*_*ej*_. The *p*-values for the tests of specific genome regions were calculated with a level of significance − Log_10_(*p*-value) = 5. Additionally, Prediction Error Variance () was employed for a second standardization, and it was called the *SNP*_*epj*_ statistic. After the analyses, we obtained higher *p*-values (maximum − Log_10_(*p*-value) = 0.20) and detected stronger signals (higher peaks in the Manhattan plot) for *SNP*_*epj*_ than with *SNP*_*ej*_. Furthermore, a simulation was carried out with the same structure of SNPs markers and animal data as in the current study, in order to compare the performance of empirical *p*-values of both standardized tests. The SNPs markers of chromosome 18 were reshuffled, and two scenarios were simulated: 1) Dependent genotypes (LD), and 2) Independent genotypes (LE). Neither scenario displayed a relationship with the phenotype, whereas both standardized tests were calculated at each scenario. The reference distribution for the *p*-values considered was the uniform. In the independent scenario (LE), standardization with  gave an empirical distribution of *p*-values that resembled the uniform density, but in the dependent scenario (LD) the *SNP*_*ej*_ performed conservatively. Instead, the standardization with  produced conservative results in the independent scenario (LE), and very conservative tests in the dependent scenario (LD). In this context, standardizing SNP effects with  resulted in *p*-values that were closer to the simulated ones. Moreover, the performance of *SNP*_*ej*_ under LD was not too conservative, a scenario that could be extrapolated to the genotypes in the current study. In addition, the *p*-values calculated using the EMMA procedure [24] were similar to those obtained with *SNP*_*ej*_. These results suggest that *SNP*_*ej*_ behaves reasonably to control type I error rate or false positives. Also, the computing time for fitting model (2a) and then calculating (6) using expressions (3)-(5) was 2.5 to 3 times less than the computing time for the EMMA model.

In order to identify SNP with important phenotypic associations [34], the calculation of SNP effects  from genomic breeding values  [8, 9, 34] has been used in several studies [5, 20, 21]. In this context, the variance of SNP effects has been estimated using different approaches. Wang et al. [21] employed the classical definition of the variance of additive effects from quantitative genetics [35], so that the variance for each *j*th marker was obtained as follows: . Whereas, McClure et al. [20] proposed equating the variance of SNP effects to , and then normalizing the SNP effects with the square root of this estimated and constant variance. This test performed similar to *SNP*_*ep j*_ (7), when the estimated SNP effects  was divided by a constant denominator, a value almost equal to the prior variance 2.67, and resulted in a very conservative test.

In contrast, the advantage of the standardized test (*SNP*_*ej*_) presented here was that each SNP effect was scaled by its own (and different) standard deviation rather than the use of a prior variance [20] or by the square of each specific SNP effect  [21] as variance. Furthermore, the computation of *SNP*_*ej*_, involves the same variance for the same SNPs markers and animals, i.e. , and the use of the standardized incidence matrix ***Z***, a function of 2*p*_*j*_(1 − *p*_*j*_), takes into account this latter quantity into *SNP*_*ej*_. Additionally, the matrix ***Z*** uses the allele frequencies from the F_0_ generation calculated with unrelated individuals, and a proper expected variance by marker (see Methods section). In addition, the test statistics *SNP*_*ej*_ that standardizes SNP effects produces a *p*-value, a result that is appealing to many researchers that are more familiar with the method of testing one SNP at the time rather than with the proportion of additive variance that is explained by a genomic region. A further advantage of the method is that detection of many false positives are avoided, and genome positions with sizeable effects are highlighted.

### Candidate segment approach

Later in the research, genome segments that expressed higher signals were located. To this purpose, SNPs with the smallest *p*-values from *SNP*_*ej*_ (6) were selected, and for each of these SNP a segment of 2 Mb long (1 Mb at each side) was created. The next step was to estimate the variance components and the Log-Likelihood from the centered animal models (2a) and (8). The latter model includes the random vector of SNP segments ***a***_1_. Lastly, we compare the performance of both models. Hayes et al. [4] used a similar model to (8), although the random SNP effect was taken from the breeding value and fitted as a separate segment effect. We observed similar results from the use of either approach. The advantage of fitting model (8) is that matrix ***G*** is the same for all segments, so that it was calculated only once, and stored in memory for the calculations, whereas in the model of Hayes et al. [13] a different ***G*** has to be calculated for each segment. This implies an extended computing time and higher requirements of CPU memory to obtain similar results to those from model (8).

To evaluate the significance of the segments, the effects of each chromosome segment were tested by the Likelihood Ratio Test. The size of the test was adjusted by the Bonferroni correction. As a result, the segment located on chromosome 6 (physical position 135 Mb-137 Mb) was significant, and explained 11% of the trait total variance. Previous studies by Edwards et al. [10] and Choi et al. [36], using microsatellites and a small number of SNP, found significant regions (physical positions between 135 and 139 Mb) on chromosome 6 for *13 week tenth rib backfat* in the current population under study.

Additionally, forty eight markers between the physical position between 128 Mb and 139 Mb on chromosome 6 (http://www.animalgenome.org/QTLdb/pig.html), have been reported to be associated with the trait. Furthermore, recent studies showed the importance of chromosome 6 [37, 38] in the expression of the trait. Therefore, our results confirm the presence of genetic variability in the trait from chromosome 6.

## Conclusions

Fast genome screening of SNP effects linearly transformed from genomic breeding values is advantageous, as a by-product of genomic evaluations for different species of farm animals. Moreover, the standardized tests of SNP effects using their own variance  developed in this study helps in detecting specific genomic regions involved in the additive variation of the trait and reducing false positive locations using less computing time. Additionally, genome segments of about 2 Mb formed by surrounding the SNP with the smallest *p*-values on each chromosome, and tested with a standardized test involving  and with the Bonferroni correction, could detect genome regions responsible for sizeable fractions of the trait genetic variance. This methodology involving genome scan and candidate segment approach is a useful method for meta-analyses of genome-wide association studies, as it enables the detection of specific genome regions that affect an economically relevant trait when using multiple populations. Code and data to obtain and reproduce the results presented is publicly available at https://www.msu.edu/~steibelj/JP_files/GBLUP.html.

## Electronic supplementary material

Additional file 1: **Highest − Log**_**10**_ **(p-values) on each chromosome for trait 13-week tenth rib backfat (mm) by standardization SNP**_**ej**_ **and EMMA.** The blue and red circle represents highest − Log_10_(*p*-values) on each chromosome by the standardization *SNP*_*ej*_ and efficient mixed-model association (EMMA) using rrBLUP. respectively. (PDF 29 KB)

Additional file 2: **Dispersion plot of − Log**_**10**_ **(p-values) for trait 13-week tenth rib backfat (mm) by EMMA and standardization SNP**_**ej**_ **.** Dispersion plot for 44055 –log_10_ (*p*-values) by efficient mixed-model association (EMMA) using the rrBLUP R package in the *x* axis, and by the standardization *SNP*_*ej*_ in the *y* axis. Red straight line is the reference line 0–1. (PDF 255 KB)

Additional file 3: **Variance components and LogLikehood for models with or without the segment for all chromosomes.** (Results for the 18 chromosomes). (PDF 181 KB)
