# Supplementary figures and images for: Rapid screening for phenotype-genotype associations by linear transformations of genomic evaluations
Source: BMC Bioinformatics. 2014 Jul 19;15(1):246. doi: 10.1186/1471-2105-15-246 (PMC4112210; doi:10.1186/1471-2105-15-246)

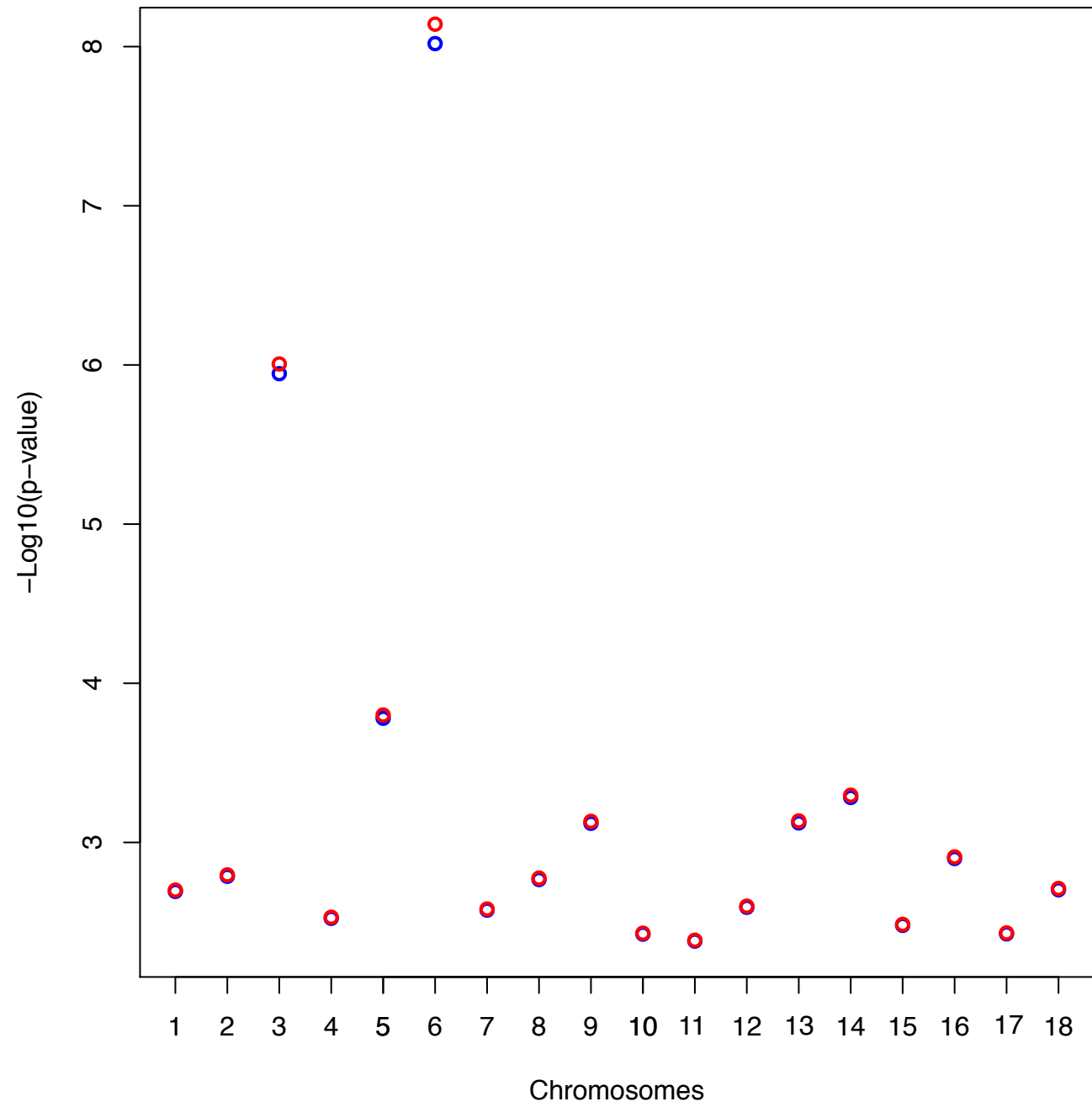

Supplement: Supplementary file 1 — Additional file 1: Highest − Log 10 (p-values) on each chromosome for trait 13-week tenth rib backfat (mm) by standardization SNP ej and EMMA. The blue and red circle represents highest − Log10(p-values) on each chromosome by the standardization SNP ej and efficient mixed-model association (EMMA) using rrBLUP. respectively. (PDF 29 KB) [file 12859_2014_6514_MOESM1_ESM.pdf]

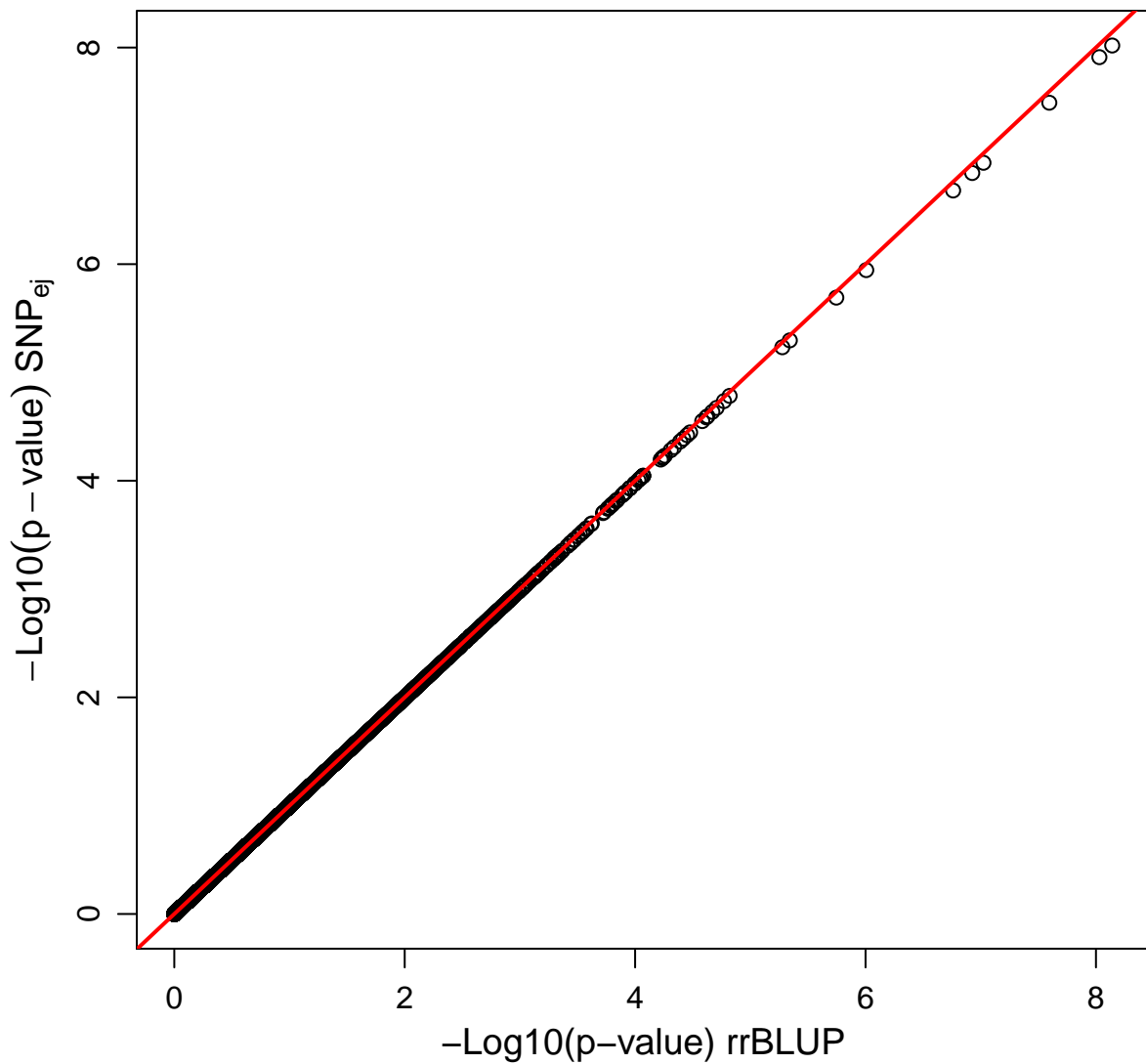

Supplement: Supplementary file 2 — Additional file 2: Dispersion plot of − Log 10 (p-values) for trait 13-week tenth rib backfat (mm) by EMMA and standardization SNP ej . Dispersion plot for 44055 –log10 (p-values) by efficient mixed-model association (EMMA) using the rrBLUP R package in the x axis, and by the standardization SNP ej in the y axis. Red straight line is the reference line 0–1. (PDF 255 KB) [file 12859_2014_6514_MOESM2_ESM.pdf]
