# Supplementary material for: Rapid screening for phenotype-genotype associations by linear transformations of genomic evaluations
Source: BMC Bioinformatics. 2014 Jul 19;15(1):246. doi: 10.1186/1471-2105-15-246 (PMC4112210; doi:10.1186/1471-2105-15-246)
Supplement: Supplementary file 3 — Additional file 3: Variance components and LogLikehood for models with or without the segment for all chromosomes. (Results for the 18 chromosomes). (PDF 181 KB) [file 12859_2014_6514_MOESM3_ESM.pdf]

Additional files

Additional file 1 – Variance components and LogLikelihood for models with or without the segment for all chromosomes

Seg-chromosome	6	3	5	14	13	9	16	2
**$-Log_{10}$(p-value)**	8.02	5.94	3.78	3.28	3.12	3.12	2.90	2.79
**Lk_m1**	−1227.938	−1227.938	−1227.938	−1227.938	−1227.938	−1227.938	−1227.938	−1227.938
**Lk_m2**	−1210.800	−1223.178	−1224.540	−1227.746	−1226.184	−1228.144	−1226.223	−1226.625
**LRT**	34.28	9.52	6.80	0.38	3.51	-0.41	3.43	2.63
**$p\text{-value}_{LRT}$**	1.1×10^{-9}	6.5×10^{-4}	3.1×10^{-3}	0.3	2.4×10^{-2}	1.0	2.5×10^{-2}	4.3×10^{-2}
**VarE_m1**	3.70	3.70	3.70	3.70	3.70	3.70	3.70	3.70
**VarA_m1**	2.68	2.68	2.68	2.68	2.68	2.68	2.68	2.68
**VarE_m2**	3.73	3.67	3.69	3.70	3.71	3.69	3.65	3.68
**VarA_m2**	1.95	2.42	2.55	2.64	2.56	2.63	2.59	2.64
**segmVA**	0.70	0.63	0.15	0.06	0.10	0.27	0.26	0.14
**%segmVA**	0.11	0.09	0.02	0.01	0.02	0.04	0.04	0.02

Seg-chromosome= Number of chromosome where segment is located, **m1**= model (2a) without the segment: $\boldsymbol{y} = \boldsymbol{X}\boldsymbol{\beta} + \boldsymbol{a} + \boldsymbol{e}$, **m2** = model (8) [illegible] the segment $\boldsymbol{y} = \boldsymbol{X}\boldsymbol{\beta} + \boldsymbol{a}_1 + \boldsymbol{a}_2 + \boldsymbol{e}$, **SNP $-Log_{10}$(p-value)**= –Logarithm in base 10 of the SNP *p*-value selected to create a segment, **Lk_[illegible]** –LogLikelihood for m1, **Lk_m2**= –LogLikelihood for m2, **LRT**= Likelihood Ratio Test for m1 and m2, **$p\text{-value}_{LRT}$**= *p*-value for LRT, **VarE_[illegible]** Error variance (σ_e^2) of m1, **VarA_m1**= Additive variance (σ_A^2) of m1, **VarE_m2**= Error variance (σ_e^2) of m2, **VarA_m2**= Additive variance $(\sigma_A^2$ [illegible] m2 , **segmVA**= Additive variance segment $(\sigma_{A_1}^2)$ of m2, **%segmVa**= Proportion in % of the total variance explained by the segm[illegible]

Additional file 1 - (Continue Table) Variance components and LogLikelihood for models with or without the segment for all chromosomes

Seg-chromosome	8	18	1	12	7	4	15	17
SNP $-\log_{10}$(p-value)	2.79	2.70	2.69	2.59	2.57	2.52	2.48	2.43
Lk_m1	−1227.938	−1227.938	−1227.938	−1227.938	−1227.938	−1227.938	−1227.938	−1227.938
Lk_m2	−1227.887	−1226.018	−1225.725	−1227.612	−1226.235	−1227.240	−1226.524	−1227.020
LRT	0.10	3.84	4.43	0.65	3.41	1.40	2.83	1.84
p-value$_{LRT}$	0.48	0.02	0.01	0.22	0.03	0.11	0.04	0.08
VarE_m1	3.70	3.70	3.70	3.70	3.70	3.70	3.70	3.70
VarA_m1	2.68	2.68	2.68	2.68	2.68	2.68	2.68	2.68
VarE_m2	3.69	3.71	3.68	3.71	3.67	3.70	3.69	3.71
VarA_m2	2.65	2.56	2.58	2.60	2.64	2.71	2.64	2.57
segmVA	0.10	0.07	0.13	0.06	0.11	-0.02	0.08	0.07
%segmVA	0.02	0.01	0.02	0.01	0.02	0.00	0.01	0.01

Seg-chromosome= Number of chromosome where segment is located, **m1**= model (2) without the segment: $\boldsymbol{y} = \boldsymbol{X\beta} + \boldsymbol{a} + \boldsymbol{e}$, **m2** = model (7) wit the segment $\boldsymbol{y} = \boldsymbol{X\beta} + \boldsymbol{a}_1 + \boldsymbol{a}_2 + \boldsymbol{e}$, **SNP $-\log_{10}$(p-value)**= –Logarithm in base 10 of the SNP *p*-value selected to create a segment, **Lk_m1** –LogLikelihood for m1, **Lk_m2**= –LogLikelihood for m2, **LRT**= Likelihood Ratio Test for m1 and m2, **p-value$_{LRT}$**= *p*-value for LRT, **VarE_m1** Error variance (σ_e^2) of m1, **VarA_m1**= Additive variance (σ_A^2) of m1, **VarE_m2**= Error variance (σ_e^2) of m2, **VarA_m2**= Additive varianc (σ_A^2) of m2 , **segmVA**= Additive variance segment $(\sigma_{A_1}^2)$ of m2, **%segmVa**= Proportion in % of the total variance explained by the segment.

Additional file 1- (Continue Table) Variance components and LogLikelihood for models with or without the segment for all chromosomes

Seg-chromosome	10	11
SNP $-\log_{10}$(p-value)	2.42	2.38
Lk_m2	−1226.179	−1227.640
Lk_m1	−1227.938	−1227.938
LRT	3.52	0.60
p-value$_{LRT}$	0.02	0.02
VarE_m2	3.70	3.70
VarA_m1	2.68	2.68
VarE_m2	3.73	3.70
VarA_m2	2.66	2.64
segmVA	−0.03	0.04
%segmVA	0.00	0.01

Seg-chromosome= Number of chromosome where segment is located, **m1**= model (2a) without the segment: $\boldsymbol{y} = \boldsymbol{X}\boldsymbol{\beta} + \boldsymbol{a} + \boldsymbol{e}$, **m2** = model (8) wit the segment $\boldsymbol{y} = \boldsymbol{X}\boldsymbol{\beta} + \boldsymbol{a}_1 + \boldsymbol{a}_2 + \boldsymbol{e}$, **SNP $-\log_{10}$(p-value)**= –Logarithm in base 10 of the SNP *p*-value selected to create a segment, **Lk_m1** –LogLikelihood for m1, **Lk_m2**= –LogLikelihood for m2, **LRT**= Likelihood Ratio Test for m1 and m2, **p-value$_{LRT}$**= *p*-value for LRT, **VarE_m1** Error variance (σ_e^2) of m1, **VarA_m1**= Additive variance (σ_A^2) of m1, **VarE_m2**= Error variance (σ_e^2) of m2, **VarA_m2**= Additive varianc (σ_A^2) of m2 , **segmVA**= Additive variance segment $(\sigma_{A_1}^2)$ of m2, **%segmVa**= Proportion in % of the total variance explained by the segment.
